# Functional Recruitment of Human Complement Inhibitor C4b-Binding Protein to Outer Membrane Protein Rck of *Salmonella*

**DOI:** 10.1371/journal.pone.0027546

**Published:** 2011-11-10

**Authors:** Derek K. Ho, Jorma Tissari, Hanna M. Järvinen, Anna M. Blom, Seppo Meri, Hanna Jarva

**Affiliations:** 1 Infection Biology Program, Department of Bacteriology and Immunology, Haartman Institute, University of Helsinki, Helsinki, Finland; 2 Division of Medical Protein Chemistry, Lund University Department of Laboratory Medicine, Malmö, Sweden; 3 HUSLAB, Helsinki University Central Hospital, Helsinki, Finland; Indian Institute of Science, India

## Abstract

Resistance to complement mediated killing, or serum resistance, is a common trait of pathogenic bacteria. Rck is a 17 kDa outer membrane protein encoded on the virulence plasmid of *Salmonella enterica* serovars Typhimurium and Enteritidis. When expressed in either *E. coli* or *S. enterica* Typhimurium, Rck confers LPS-independent serum resistance as well as the ability to bind to and invade mammalian cells. Having recently shown that Rck binds the inhibitor of the alternative pathway of complement, factor H (fH), we hypothesized that Rck can also bind the inhibitor of the classical and lectin pathways, C4b-binding protein (C4BP). Using flow cytometry and direct binding assays, we demonstrate that *E. coli* expressing Rck binds C4BP from heat-inactivated serum and by using the purified protein. No binding was detected in the absence of Rck expression. C4BP bound to Rck is functional, as we observed factor I-mediated cleavage of C4b in cofactor assays. In competition assays, binding of radiolabeled C4BP to Rck was reduced by increasing concentrations of unlabeled protein. No effect was observed by increasing heparin or salt concentrations, suggesting mainly non-ionic interactions. Reduced binding of C4BP mutants lacking complement control protein domains (CCPs) 7 or 8 was observed compared to wt C4BP, suggesting that these CCPs are involved in Rck binding. While these findings are restricted to Rck expression in *E. coli,* these data suggest that C4BP binding may be an additional mechanism of Rck-mediated complement resistance.

## Introduction

Any successful human pathogen must possess mechanisms for resisting complement, a key first-line defense of the innate immune system. The complement system consists of approximately 40 proteins found in the fluid phase and on cell surfaces. Upon recognition of an invader, this system is immediately activated via one or several routes: the classical, lectin, or alternative pathways, which all converge at the C3 step. Successful activation on a pathogen surface leads to opsonization with C3b and its cleavage product iC3b. Complement activation also results in generation of inflammation via the released anaphylatoxins, and in the case of gram-negative bacteria, direct lysis by the membrane attack complex (MAC) [1]. Accordingly, pathogenic bacteria have evolved effective mechanisms for evading or resisting complement attack [2].

Serum resistance, or resistance to complement-mediated killing, is a recognized virulence trait of *Salmonella*. Several diverse mechanisms of serum resistance have been described, including prevention of functional MAC insertion into the outer membrane due to long-chain LPS [3], [4], cleavage of complement components C3, C4 and C5 by PgtE [5], and C6 inhibition by TraT [6]. The outer membrane protein Rck has been shown to mediate serum resistance by interfering with MAC function [7], while the mechanism of serum resistance by the related protein, PagC, is currently unknown [8].

Within the *Enterobacteriaceae*, a family of highly conserved 17–19 kDa outer membrane proteins including Rck, PagC, OmpX (*E. coli* and *Enterobacter cloacae*), and Ail (*Yersinia*) [9] has been described. All these proteins have been shown to possess virulence associated phenotypes. Based on structural models and the solved crystal structure of OmpX [10], these proteins are predicted to exhibit a common topology consisting of eight transmembrane amphipathic β-strands and four surface exposed loops. The regions of greatest homology are the transmembrane domains, while the greatest sequence diversity is found in the surface exposed loops, which presumably mediate interactions with the host. This observation suggests that these proteins are functionally dissimilar. Despite the fact that Rck, Ail, and PagC have been shown to mediate serum resistance, it is not currently clear if these proteins do so by conserved mechanisms.

Although the complement system plays a critical role in host defense against infection, inappropriate or excessive activation can lead to damage of self tissues. To ensure that complement activation is restricted to the appropriate targets, the host has evolved multiple cell surface and fluid phase inhibitors which regulate complement at the steps of activation, amplification, and membrane attack [11]. Not surprisingly, numerous pathogenic microbes have evolved the ability to recruit and exploit fluid-phase complement inhibitors as a means of defense against complement attack [2]. Particularly attractive targets for pathogens include factor H (fH) and C4b-binding protein (C4BP), which are fluid-phase inhibitors of the alternative (AP) and the classical (CP) and the lectin pathway (LP), respectively. Functional recruitment of one or both of these proteins would confer the ability to inhibit complement activation at the convertase step, thus preventing complement-mediated opsonization, inflammation, and lysis via MAC.

C4BP is a key fluid phase inhibitor of the CP and LP. It is a large glycoprotein with a plasma concentration of approximately 200 µg/ml. The major isoform found in plasma consists of 7 identical α chains and a single β chain. The α and β chains consist of 8 and 3 complement control protein domains (CCPs), respectively. C4BP regulates CP and LP mediated complement activation by three mechanisms: by acting as a cofactor for factor I (fI)-mediated cleavage and inactivation of C4b, by accelerating the decay of the C4b2a convertase, and by preventing the assembly of the convertase by binding to C4b [12]. Recruitment of C4BP as a means of defense against complement attack has been demonstrated in several bacterial pathogens such as *N. gonorrhoeae* [13], *E. coli* K1 [14], *S. pneumoniae* [15], *B. burgdorferi* sensu lato [16] and *H. influenzae* [17].

The Rck homologue in *Y. enterocolitica*, Ail, has been shown to functionally recruit both fH [18] and C4BP [19]. Based on these observations, we hypothesized that Rck possesses a similar ability. We have recently shown that Rck binds and recruits fH in a functional manner, and can protect serum-sensitive *E. coli* from AP-mediated killing [20]. We have extended these observations and demonstrate here that Rck can additionally bind the CP and LP inhibitor C4BP. This binding is functional and specific, and appears to involve CCPs 7 and 8 of C4BP. These results suggest that Rck has the ability to functionally recruit multiple complement inhibitors, thus conferring the ability to resist attack from this key arm of innate immunity.

## Materials and Methods

### Ethics statement

All persons who donated blood for this study provided a written informed consent. The study protocol has been approved by the Section for Research of the Helsinki University Central Hospital Laboratory (project TYH7214).

### Bacterial plasmids, strains and growth

Serum-sensitive *E. coli* strain BL21(DE3) (Invitrogen, Carlsbad, CA) was used for all experiments. Bacteria were grown in Luria-Bertani (LB) broth cultures with shaking or on solid LB media at 37°C in room air. Plasmid pRck was used to express Rck (GenBank: M76130.1) in *E. coli* BL21(DE3). This plasmid contains the *rck* gene PCR amplified from the virulence plasmid of *Salmonella enterica* serovar Typhimurium strain SL1344 and was cloned into plasmid pBR322. Bacteria containing pRck or pBR322 were cultured in the presence of ampicillin (100 µg/ml). pRck was a kind gift from Dr. Nobuhiko Okada (Kitasato University, Tokyo, Japan) and has been described previously [8].

### Sera, proteins and antibodies

Normal human serum (NHS) was pooled from blood collected from 7 to 10 healthy adult laboratory personnel with written informed consent. The study protocol has been approved by the Section for Research of the Helsinki University Central Hospital Laboratory (project TYH7214). The blood was then allowed to clot and the serum was subsequently harvested, pooled, aliquoted and stored at −70°C until used. Heat-inactivated serum (HIS) was generated by incubating NHS for 1 h at 56°C. Purified human C4b and factor I were purchased from Calbiochem (San Diego, CA). Human C4BP was purified according to the protocol of Persson [21]. Bovine serum albumin and heparin were purchased from Sigma-Aldrich. Single CCP deletion mutants of human C4BP were purified as described [22]. Polyclonal rabbit anti-human C3c (which recognizes the C3c portion of C3b, iC3b and native C3) and monoclonal mouse anti-human C5b-9 (Dako, Glostrup, Denmark), monoclonal mouse anti-human C4BP MK104 [22], monoclonal mouse anti-human C4BP (Quidel, San Diego, CA), and sheep polyclonal anti-human C4BP (The Binding Site, Birmingham, UK) were used as primary antibodies in flow cytometry experiments. The appropriate Alexa Fluor labeled secondary antibodies were acquired from Invitrogen.

### Direct C4BP binding assays

Bacteria were grown to stationary phase in 5 ml broth cultures O/N using disposable 16×125 mm tubes (BD Biosciences, San Jose, CA). Thereafter, they were washed and resuspended in Veronal buffered saline (142 mM NaCl, 1.8 mM sodium barbital, 3.3 mM barbituric acid, pH 7.4–7.6) supplemented with 0.1% gelatin (GVB) to a final concentration of 1×10^9^ CFUs/ml. 20 µl of this solution was then incubated with 20 µl of ^125^I-C4BP (∼ 20,000 cpm/sample) for 30 min at 37°C with agitation. After incubation, the samples were centrifuged through 250 µl of 20% sucrose/GVB at 10,000 x g to separate free protein from protein bound to the bacteria. The supernatants and pellets were separated and radioactivities were measured in a gamma counter. The ratio of bound to total radioactivity was then determined. Competition assays were performed by determining the relative binding of ^125^I-C4BP in the presence of increasing amounts of unlabeled C4BP, heparin, BSA, or NaCl.

### Flow Cytometry

Bacteria grown as described above were centrifuged at 10,000 x g for 3 min and resuspended in Dulbecco's phosphate buffered saline (DPBS) to a final OD_600_ of 0.4 (4×10^8^ CFU/ml). 25 µl of the bacterial suspension was added to HIS or NHS (final concentration specified for each experiment) or mixed with purified human C4BP in DPBS (final concentration specified in each experiment) to a final volume of 50 µl. Samples were incubated at 37°C for the indicated times. The samples were removed, centrifuged, and washed three times in 50 µl DPBS supplemented with 1% BSA. After the final wash, bacteria were resuspended in 50 µl DPBS/1% BSA. 20 µl of a 1∶100 dilution of the appropriate primary antibody (diluted in DPBS) was added to the bacteria (final volume 70 µl) and incubated at room temperature for 20 min. After washing in DPBS, bacteria were resuspended in 50 µl DPBS, to which 20 µl of 1∶200 dilution of the appropriate Alexa Fluor 488-conjugated secondary antibody was added, followed by incubation at room temperature in the dark for 20 min. The cells were washed twice as above and resuspended in 0.5 ml filtered DPBS containing 1% paraformaldehyde (Electron Microscopy Sciences, Hatfield, PA). Flow cytometric analysis was performed on a FACscan (BD Biosciences) or Cyan ADP cytometer (Beckman Coulter, Miami, FL).

### Cofactor activity for C4b cleavage

Bacteria grown as described above were resuspended to a final concentration of 1×10^9^ CFU/ml in DPBS. The bacteria were then incubated with purified human C4BP at a final concentration of 5 µg/ml. Following a 30 min incubation at 37°C, the bacteria were washed five times in DPBS. After the last wash, bacteria were resuspended in DPBS and incubated with approximately 100,000 cpm of ^125^I-C4b and fI (15 µg/ml). After a 1 h incubation at 37°C, the samples were centrifuged and the supernatants analyzed by SDS-PAGE under reducing conditions. The gels were subsequently dried and the results visualized by autoradiography.

### Statistical Analysis

Where appropriate, standard deviation (SD) and/or Student's t-test was performed for data comparison and assessment of statistical significance.

## Results

### Rck binds to C4b-binding protein

**Figure 1:**
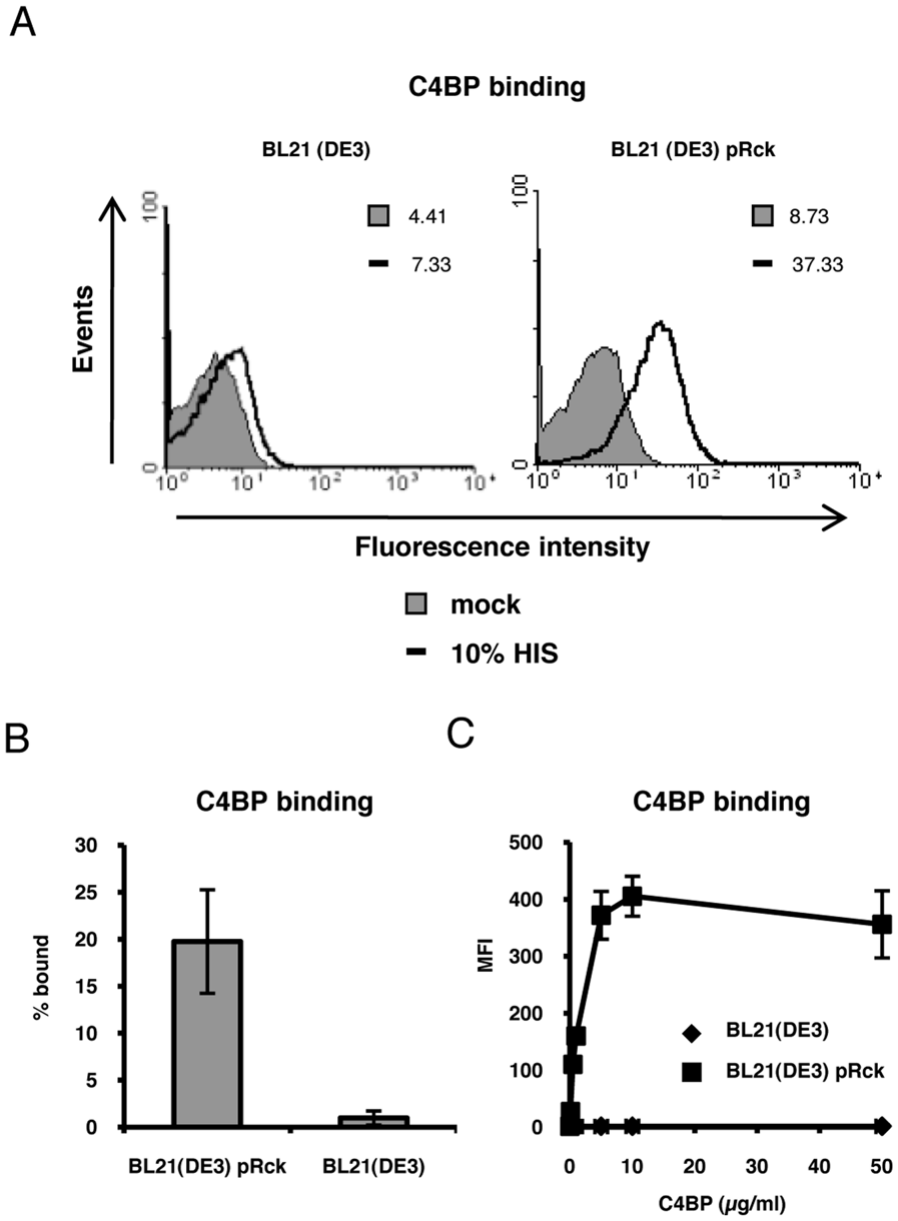
Rck is a C4BP binding protein. A, FACS analysis of C4BP binding. 2×10^8^ CFU/ml of BL21(DE3) and BL21(DE3) pRck in DPBS were mock treated (DPBS only) or incubated with 10% HIS for 30 min. C4BP binding was measured by FACS using a mouse monoclonal anti-human C4BP antibody. Mean fluorescence intensity (MFI) values are indicated. Representative data of an experiment performed at least three times are shown. B, Direct binding of ^125^I-labeled C4BP to Rck. 1×10^9^ CFU/ml of BL21(DE3) and BL21(DE3) pRck in GVB were incubated with ^125^I-C4BP (∼ 20,000 cpm/sample). The ratio of bound to total radioactivity was then determined (% bound). Data is expressed as means of two independent experiments performed in duplicate ± SD. C, Dose-dependent and saturable binding of C4BP to Rck. Bacteria prepared as in Figure 1A were incubated with increasing amounts of purified C4BP and detected by FACS as described above using a polyclonal anti-C4BP antiserum. Data is expressed as means of the MFI values acquired from three independent experiments ± SD.

In a previous study we observed that Rck expressed in *E. coli* BL21 (DE3) binds the fluid-phase inhibitor of the alternative pathway, fH [20]. The Rck homologue in *Yersinia enterocolitica,* Ail, has been shown to bind C4BP [19]. We thus decided to test the possibility that Rck also possesses this ability. As shown in Figure 1A, we observed by FACS analysis that Rck expressed in *E. coli* BL21 (DE3) can bind C4BP from HIS. This binding was not was observed on the empty strain (Figure 1A) or on *E. coli* BL21 (DE3) expressing the empty vector, pBR322 (data not shown). These results were corroborated in a direct binding assay using radiolabeled C4BP (Figure 1B). Binding of C4BP to Rck was dose-dependent and saturable, as addition of increasing amounts of C4BP resulted in increased binding to Rck, reaching a plateau at approximately 10 µg/ml (Figure 1C). No binding was observed in the absence of Rck, even at the highest concentration of C4BP used. Taken together, these results indicate that Rck binds C4BP.

### C4BP bound to Rck exhibits functional activity

**Figure 2:**
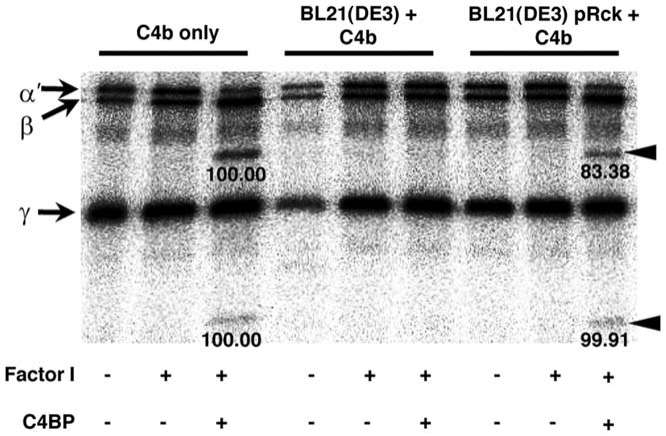
C4BP bound to Rck is functional for fI-mediated C4b cleavage. Bacteria grown as in Figure 1B were incubated with purified C4BP (5 µg/ml) for 30 min at 37°C and washed, after which fI (15 µg/ml) and ^125^I-C4b (100,000 cpm) were added. After a 1h incubation at 37°C, samples were analyzed with reducing SDS-PAGE and visualized by autoradiography. The α', β and γ chains of C4b and the C4b cleavage products are indicated by arrows and arrowheads, respectively. Densitometry values are shown beneath each cleavage product. The band intensities for the C4b only control were set to 100 and used as a reference for the corresponding band obtained from the cofactor assay performed in the presence of Rck. Representative data of an experiment performed three times is shown.

A cofactor assay was employed to determine if C4BP bound to Rck is functional. In this assay, Rck-expressing bacteria are incubated with purified C4BP and washed, followed by addition of C4b and fI. As shown in Figure 2, incubation of purified C4BP with C4b and fI results in the generation of the C4d and C4c cleavage products. These cleavage products were also observed when Rck-expressing bacteria were incubated with C4BP, washed, and then incubated with C4b and fI. No cleavage was observed in the absence of fI, suggesting that the bacteria do not possess intrinsic cofactor activity. No cleavage was observed under any conditions in the absence of Rck expression. Collectively, these results suggest that C4BP bound to Rck is functional for fI-mediated cleavage of C4b.

### Reduced C3b and MAC deposition in the presence of Rck

**Figure 3:**
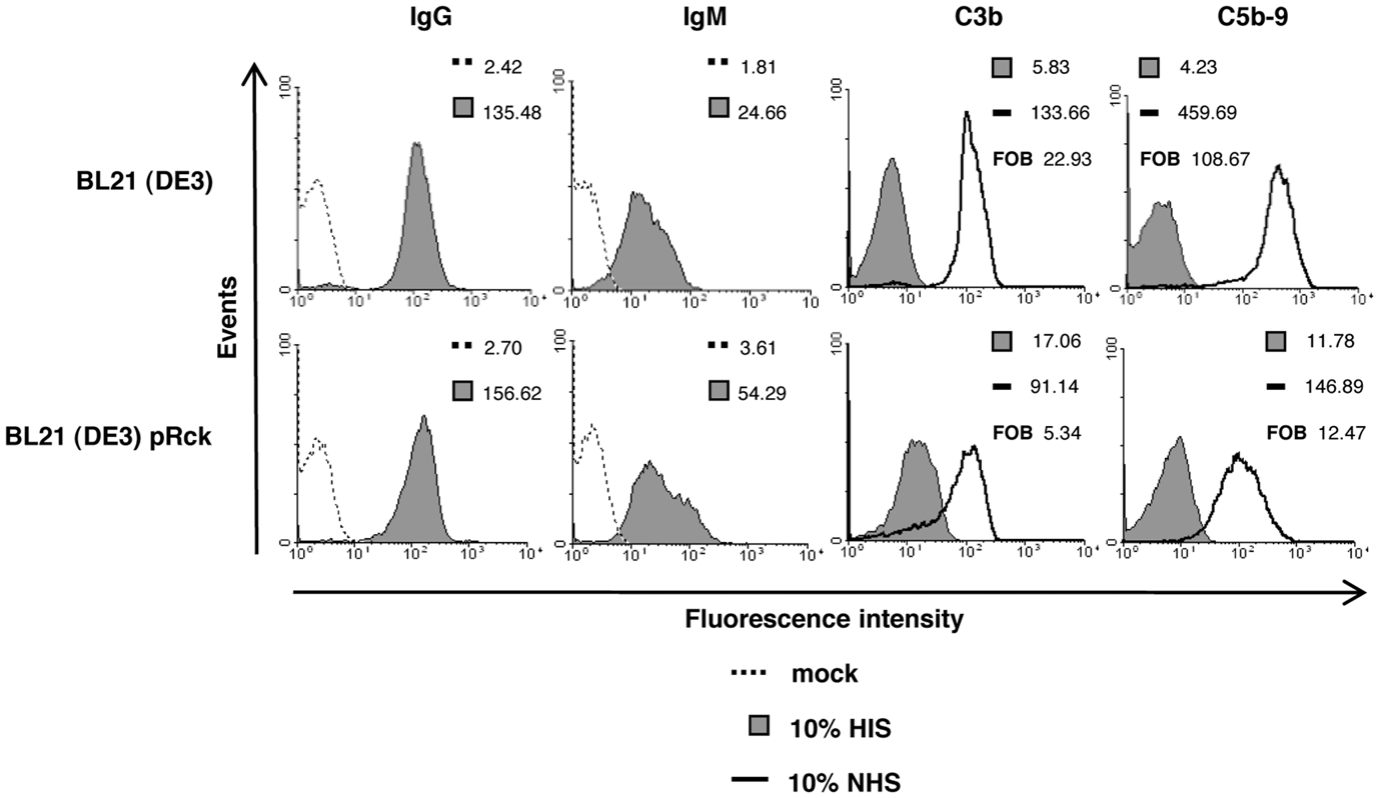
Deposition of IgG, IgM, C3b and MAC. Bacteria prepared as in Figure 1A were mock treated with DPBS (no serum) or incubated with 10% HIS or 10% NHS for 10 min at 37°C. IgG and IgM binding was measured by FACS analysis using goat anti-human IgG antibodies and rabbit anti-human IgM, respectively. Bound C3b was detected using a polyclonal rabbit anti-human C3c antibody, which recognizes the C3b, iC3b and native C3 forms. MAC deposition was detected by a mouse-monclonal antibody specific for the C5b-9 complex. MFI and FOB (fold over background, the value obtained when the MFI value from 10% NHS is divided by the corresponding value from 10% HIS) values are indicated. Representative data of an experiment performed three times is shown.

Previous investigations have demonstrated that Rck expression in *Salmonella* or *E. coli* confers resistance to both NHS and AP-only serum [7], [20]. As C4BP is an inhibitor of the classical pathway, we wanted to test the possibility that Rck is able to inhibit C3b and MAC deposition on the bacterial surface in the presence of all C pathways. We first tested binding of IgG and IgM to the bacterial surface, which is required for initiation of the CP. By FACS analysis, we observed measurable IgG and IgM binding from HIS on the bacterial surface regardless of the presence or absence of Rck (Figure 3). We then tested the ability of Rck to inhibit C3b and MAC deposition. Following a 10 minute incubation in 10% NHS, we observed reduced C3b and MAC deposition on bacteria expressing Rck compared to the empty strain (Figure 3). As we observed higher levels of background on the Rck expressing strain while measuring C3b and MAC (perhaps due to primary antibody cross-reactivity with Rck), the data is also expressed as fold over background (FOB), which is obtained by dividing the MFI value from 10% NHS with the corresponding value from 10% HIS. Taken together, these results suggest that the CP is active on the strains employed and that Rck is capable of inhibiting C3b and MAC in the presence of all C pathways.

### C4BP binding competition assays

**Figure 4:**
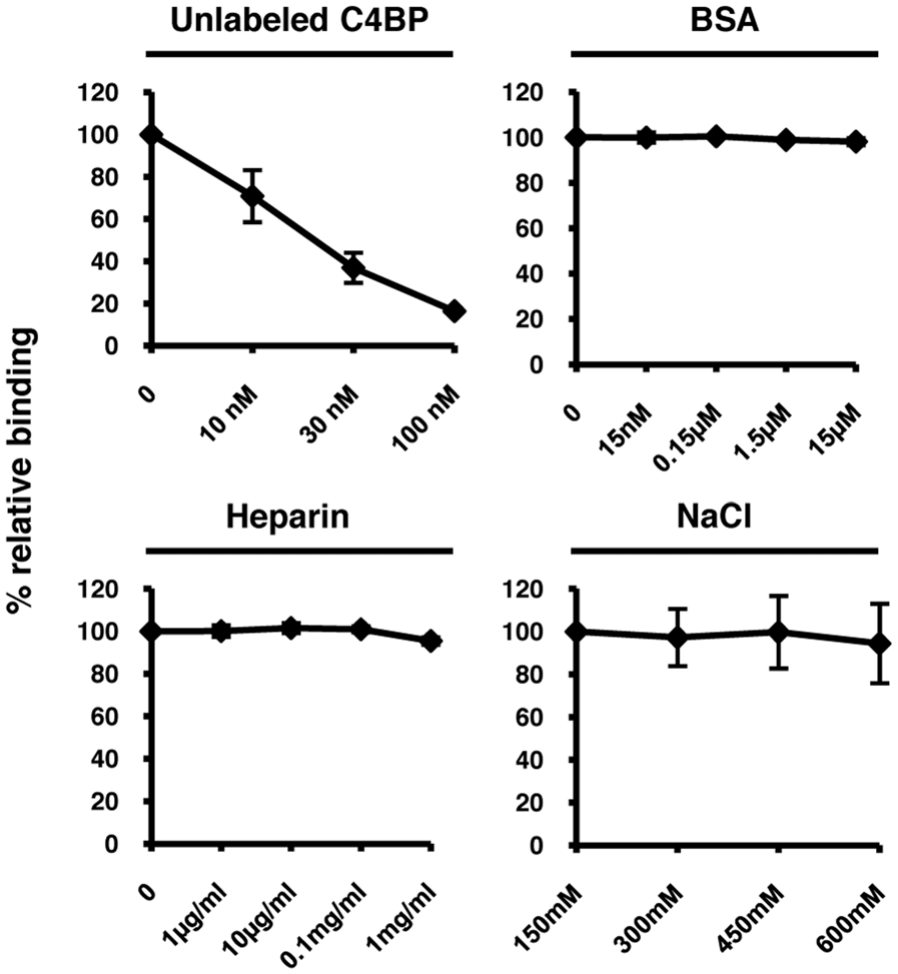
Competition experiments. Characterization of C4BP binding to Rck. Direct binding of C4BP as performed in Figure 1B in the presence of increasing concentrations of unlabeled C4BP, bovine serum albumin (BSA), heparin, or NaCl. Relative binding indicates the amount of C4BP bound in the absence of competitor (set to 100%) compared to the amount bound in the presence of competitor. Data are shown as means of at least four independent experiments ±SD.

To determine the specificity of the interaction between C4BP and Rck, we used either unlabeled C4BP or BSA as competitors to the binding of radiolabeled C4BP. As shown in Figure 4, we observed a dose-dependent reduction of radiolabeled C4BP binding in the presence of increasing concentrations of unlabeled C4BP, while increasing concentrations of BSA had no effect. These results suggest that the interaction between Rck and C4BP is specific. We further characterized the Rck-C4BP interaction by testing the effects of heparin and NaCl. Increasing concentrations of heparin or NaCl had no effect on binding of radiolabeled C4BP to Rck, suggesting that C4BP binds to Rck via CCPs outside of the heparin binding site (CCPs 1-3) and that the binding is mediated by non-ionic interactions.

### Mapping of the Rck binding site on C4BP

**Figure 5:**
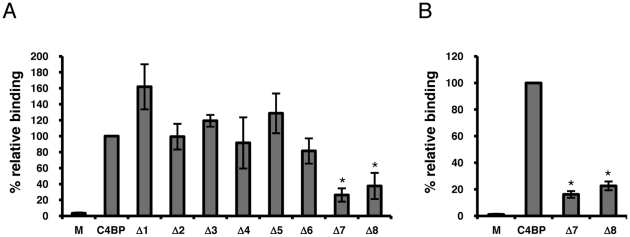
Localization of the Rck binding site on C4BP. A. BL21(DE3) pRck grown as in Figure 1A were mock treated (DPBS only) or incubated with purified C4BP or recombinant C4BP deletion mutants (10 µg/ml), each with a single individual CCP deletion (Δ1-8). Bound C4BP (wt or mutant) was detected with sheep polyclonal anti-human C4BP antibodies. The MFI value obtained from wt C4BP was set to 100% and used as a reference in comparison to the MFI values obtained for all other samples. Data are shown as means of three independent experiments ±SD. *, *p*<0.01, compared to wt C4BP. B. Detection of bound C4BP and CCP deletions 7 and 8 with MK104. Same experiment as A using MK104, which is specific for CCP1 of C4BP. *, *p*<0.01, compared to wt C4BP.

To identify the Rck binding site(s) on C4BP, we incubated bacteria expressing Rck with purified human C4BP or with equal amounts of recombinant C4BP mutants, each deficient in an individual CCP. Amounts of bound protein were determined with a polyclonal anti-human C4BP antibody followed by FACS analysis. The MFI value obtained from wt C4BP bound to Rck was set to 100% and the relative binding of the C4BP mutants determined. As shown in Figure 5A, we observed a reduction in binding of Rck to C4BP deficient in CCPs 7 and 8 (approximately 70% and 60% reduction, respectively). Due to the possibility that the polyclonal anti-C4BP antibody employed in this experiment may preferentially detect the C-terminal CCPs (such as CCPs 6-8), we also measured binding of wt C4BP and the CCP7 and 8 mutants with monoclonal anti-C4BP antibody 104 (MK104), which is specific for CCP1 of the α-chain. Consistent with the results obtained with the polyclonal antibody, using MK104 we observed an approximate 84% and 78% reduction in binding to C4BP deficient in CCPs 7 and 8, respectively (Figure 4B). Taken together, these results suggest that C4BP CCPs 7 and 8 may be responsible for the interaction with Rck. As the heparin binding sites on C4BP are located on CCPs 1-3 [23], the inability of heparin to compete with the C4BP-Rck interaction (Figure 4) is consistent with these results.

## Discussion

In this work we demonstrate that the *Salmonella* outer membrane protein Rck, when expressed in a normally serum-sensitive *E. coli* host, can bind C4BP in a functional manner. This work extends our previous observations that Rck can functionally recruit the key fluid-phase inhibitor of the AP, fH [20]. C4BP binding is specific and appears to involve CCPs 7 and 8 of C4BP. Collectively, these results suggest that fH and C4BP recruitment play an important role in Rck-mediated serum resistance.

The ability to inhibit multiple points in the complement pathway likely increases the chances of a pathogen to escape complement attack. This is demonstrated by the growing list of bacterial pathogens which possess the ability to bind multiple complement inhibitors [24]. In light of the results presented here, Rck joins a growing list of proteins with the ability to bind both C4BP and fH. Based on sequence comparisons of homologous proteins Ail, Rck and PagC, which indicate high sequence similarity only in the membrane-spanning domains, it was initially proposed that these proteins may be structurally similar but functionally distinct [25]. The observation that Ail and Rck both bind fH and C4BP (although with different sensitivities to salt and heparin and different binding regions in fH) suggests the possibility that these proteins may possess a structure particularly suited to recruitment of these complement inhibitors.

Using a panel of C4BP mutants deficient in single CCPs of the α-chain, we observed a reduction in Rck binding with C4BP mutants lacking either CCP 7 or 8 (Figure 5). This result suggests that CCPs 7 and 8 are important for the interaction with Rck, and that there may be synergy between the two neighboring domains. As the heparin binding sites in C4BP are found in CCPs 1-3, this result was consistent with our observation that increasing heparin concentrations had no effect on the C4BP-Rck interaction. A survey of other bacterial pathogens which recruit C4BP reveals that the N-terminal CCPs (CCPs 1-3) are important for interaction with the M proteins of *S. pyogenes* [26], [27] and the porin proteins from *N. meningitidis* [28] and *N. gonorrhoeae* [13]. Both the N-terminal and the C-terminal regions are involved in the interaction with OmpA of *E. coli* K1 (CCPs 3 and 8) [14] and with an unknown ligand of *H. influenzae* (CCPs 2 and 7) [17]. Collectively, these results suggest that the N and C terminal, but not central CCPs, are preferred for bacterial recruitment and exploitation. In this regard, Rck is similar to other bacterial ligands for C4BP, although there is currently no evidence to suggest structural similarities between Rck and the ligands mentioned above.

In our C4BP binding assays we observed that incubation of Rck-expressing bacteria with 1µg/ml purified human C4BP resulted in greater C4BP binding compared to 10%NHS, which contains approximately 20µg/ml C4BP (Figure 1A). This result may be due to differences in affinity of the antibodies used; binding of purified C4BP was determined using a polyclonal antiserum while a monoclonal antibody was employed for detection from HIS. Accordingly, the polyclonal antiserum may be capable of simultaneously detecting multiple C4BP epitopes, thus leading to increased fluorescence. Alternatively, although more C4BP is present at this concentration of NHS, it is possible that C4BP may be interacting with other serum components, thereby reducing its availability to bind Rck. Conversely, it may also be possible that other serum components are interacting with Rck and blocking C4BP binding, although the observed serum resistance suggests that the amount of bound C4BP is sufficient for protection against complement attack.

Cirillo and colleagues have previously demonstrated that a point mutation in the N-terminal region of the third surface exposed loop of Rck, G118D, resulted in an approximate 1.6 log reduction in serum survival when expressed in *E. coli* [29]. We tested the possibility that this mutant would possess reduced C4BP binding ability. By FACS analysis using purified C4BP, we observed that the G118D mutation bound approximately 24-fold less C4BP compared to wt Rck, although C4BP binding was not entirely eliminated (data not shown). These results suggest that C4BP binding plays an important role in the ability of Rck to mediate serum resistance.

*E. coli* was chosen for these studies because it allows the serum resistance phenotype to be studied in the absence of other serum resistance factors in *Salmonella* which have been described. Accordingly, the results presented here can be attributed to the presence of Rck, as the background strain or the background strain expressing the empty vector cannot mediate the serum resistance phenotype. Nevertheless, the interpretation of these results is restricted to this system. It is also important to note that functional fH and C4BP binding was observed on Ail in *Y. enterocolitica* O:3 provided that O-antigen or outer core hexasaccharide were absent from LPS [18], [19]. The *E. coli* BL21(DE3) strain used in these studies has a truncated LPS with only the inner core present, due to a defective *galE* gene. Although the ability of Rck to mediate invasion of cultured eukaryotic cells and inhibit MAC has been demonstrated in both *Salmonella* and *E. coli* regardless of LPS length, [7], [25], [30], the ability to bind both fH and C4BP in an appropriate *Salmonella* background and under conditions of varying LPS length remains to be tested.

We have demonstrated that Rck is a functional C4BP binding protein when expressed in a normally serum-sensitive heterologous *E. coli* host. This work extends our previous observations demonstrating functional fH binding ability in Rck. By recruiting both C4BP and fH, Rck has the ability to inhibit all pathways of complement at the convertase step, thus preventing complement-mediated opsonization, lysis, and inflammation. These results suggest that binding of complement inhibitors is critical for Rck-mediated serum resistance.
